# Comparative Landscape Genetics of Three Closely Related Sympatric Hesperid Butterflies with Diverging Ecological Traits

**DOI:** 10.1371/journal.pone.0106526

**Published:** 2014-09-03

**Authors:** Jan O. Engler, Niko Balkenhol, Katharina J. Filz, Jan C. Habel, Dennis Rödder

**Affiliations:** 1 Zoological Research Museum Alexander Koenig, Bonn, Germany; 2 Department of Wildlife Sciences, University of Göttingen, Göttingen, Germany; 3 Department of Biogeography, Trier University, Trier, Germany; 4 Museum of Natural History Dortmund, Dortmund, Germany; 5 Department of Ecology and Ecosystemmanagement, Technical University Munich, Freising-Weihenstephan, Germany; Tuscia University, Italy

## Abstract

To understand how landscape characteristics affect gene flow in species with diverging ecological traits, it is important to analyze taxonomically related sympatric species in the same landscape using identical methods. Here, we present such a comparative landscape genetic study involving three closely related Hesperid butterflies of the genus *Thymelicus* that represent a gradient of diverging ecological traits. We analyzed landscape effects on their gene flow by deriving inter-population connectivity estimates based on different species distribution models (SDMs), which were calculated from multiple landscape parameters. We then used SDM output maps to calculate circuit-theoretic connectivity estimates and statistically compared these estimates to actual genetic differentiation in each species. We based our inferences on two different analytical methods and two metrics of genetic differentiation. Results indicate that land use patterns influence population connectivity in the least mobile specialist *T. acteon*. In contrast, populations of the highly mobile generalist *T. lineola* were panmictic, lacking any landscape related effect on genetic differentiation. In the species with ecological traits in between those of the congeners, *T. sylvestris*, climate has a strong impact on inter-population connectivity. However, the relative importance of different landscape factors for connectivity varies when using different metrics of genetic differentiation in this species. Our results show that closely related species representing a gradient of ecological traits also show genetic structures and landscape genetic relationships that gradually change from a geographical macro- to micro-scale. Thus, the type and magnitude of landscape effects on gene flow can differ strongly even among closely related species inhabiting the same landscape, and depend on their relative degree of specialization. In addition, the use of different genetic differentiation metrics makes it possible to detect recent changes in the relative importance of landscape factors affecting gene flow, which likely change as a result of contemporary habitat alterations.

## Introduction

In the theory of island biogeography, McArthur & Wilson [1] predicted the evolution of biodiversity on islands based on two key factors: habitat size and isolation. Later, this island based model was adopted to explain population structure of organisms in mainland ecosystems consisting of habitat patches surrounded by a semi- or non-permeable matrix. This mainland transformation of the theory of island biogeography inspired the fundamental paradigm of the metapopulation concept [2]–[3] and also of the neutral theory in both macroecology and population genetics [4]–[5]. Ultimately, island biogeography theory revolutionizes our thinking on habitat fragmentation and conservation biology (summarized in [6]). Apart from habitat size and isolation, spatial biodiversity patterns are also influenced by additional factors such as habitat quality [7], intrinsic characteristics of species-specific dispersal behaviour [8]–[9] and ecological tolerance [10] of species. Importantly, population responses are highly species-specific, when the quality of the landscape matrix in between suitable habitat patches is reduced [11]. This would also have consequences for global biodiversity [12]–[13] and large scale conservation efforts [14].

Understanding the effects of the landscape matrix on realized dispersal and functional population connectivity is also a major focus of landscape genetics [15]–[17]. Incorporating spatial landscape information with population genetic data goes far beyond the classical analysis of isolation-by-distance (IBD) [18]. Species respond differently to the landscape, in terms of their dispersal, which ultimately affects the rates of gene flow among local populations [19]–[20]. While the classical isolation-by-distance approach introduced by Wright [18] accounts for the geographic (Euclidean) distance between sampled populations only, other approaches such as the recently proposed isolation-by-resistance (IBR) concept [21] accounts for these species-specific responses to different landscape components that impede or favor gene-flow across a given landscape matrix.

Many studies assess landscape effects on gene flow in only a single species. However, to understand how landscape effects on gene flow differ between species, and to take effective conservation actions, it is important to analyze multiple species in the same landscape using identical methods [22]. However, past studies comparing different species mostly focused on species that inhabited comparable habitats, but were taxonomically independent [19]–[20], [23]. A different comparative approach is to analyze landscape genetic relationships in closely related taxa inhabiting the same landscape. Such a focus on taxonomically related sympatric species (i.e. congeneric species which have the same or overlapping geographic ranges, regardless of whether or not they co-occur at the same locality) allows the assessment of traits that gradually change between the congeners independently from confounding effects that may arise in relation to different evolutionary histories or environments, respectively [24]–[25]. Next to dispersal propensity, niche breadth (i.e. the degree of specialization on specific habitat traits) is a very important trait in this respect, as it is directly associated with the available habitat within a landscape.

Generalist species can be found in a broader variety of ecosystems, showing higher abundances and broader spatial distributions. In contrast, specialist species demanding certain habitat conditions are often geographically restricted to specific habitats and usually occur in lower local abundances [26]. Apart from ecological demands, connectivity among local populations is further influenced by the dispersal propensity of species. Typically, sedentary species are mostly characterized by rather limited individual exchange compared to species with strong dispersal behavior. These ecological and behavioral traits also affect the genetic structure of generalist versus specialist species [10], [26]–[27]. Organisms with specific habitat demands and restricted dispersal behavior should generally be characterized by low gene flow resulting in strong genetic differentiation. In contrast, species with weaker habitat specificity and higher dispersal propensity should show increased levels of gene flow, leading to a lack of genetic differentiation. Importantly, landscape influences on gene flow and resulting genetic patterns could also differ between generalist and specialist species inhabiting the same landscape.

In this study, we present a comparative landscape genetic analysis involving three closely-related butterfly species, to assess the impact of landscape parameters (i.e. land use, topography and climatic conditions) on the genetic structure of sympatric species with different ecological traits. We re-analyzed a molecular dataset taken from a previous study [28], where landscape effects were previously ignored, involving three congeneric, but ecologically divergent skipper species of the genus *Thymelicus* (Hubner 1890). The three species include the generalist *T. lineola*, which occurs in high abundances and shows strong dispersal propensity; the specialist *T. acteon* which is sedentary and occurs restricted to specific habitats; and *T. sylvestris*, which lies in between these two extremes in terms of habitat specificity and dispersal abilities. Using these three species, we (i) investigate the impact of ecological traits on species-specific functional landscape connectivity and (ii) determine the overall relevance of landscape characteristics for connectivity in each species, as well as the individual importance of topography, climatic conditions and land-use parameters. We hypothesized that species-specific landscape effects on gene flow should follow the cline of specialization in the three Hesperid butterflies, with strongest landscape effects on genetic differentiation in the most specialized *T. acteon* and weakest landscape effects in the generalist *T. lineola*.

## Material and Methods

### Ethics statement

The research was conducted under permission, to collect butterflies and to work in several protected areas, by the local authorities of Saarbrücken (Germany, Saarland), Koblenz (Germany, Rhineland-Palatinate), Luxembourg, and Metz (Loraine, France). Imagoes of the respective species were stored in liquid nitrogen until genetic analysis.

### Study area and species

**Figure 1:**
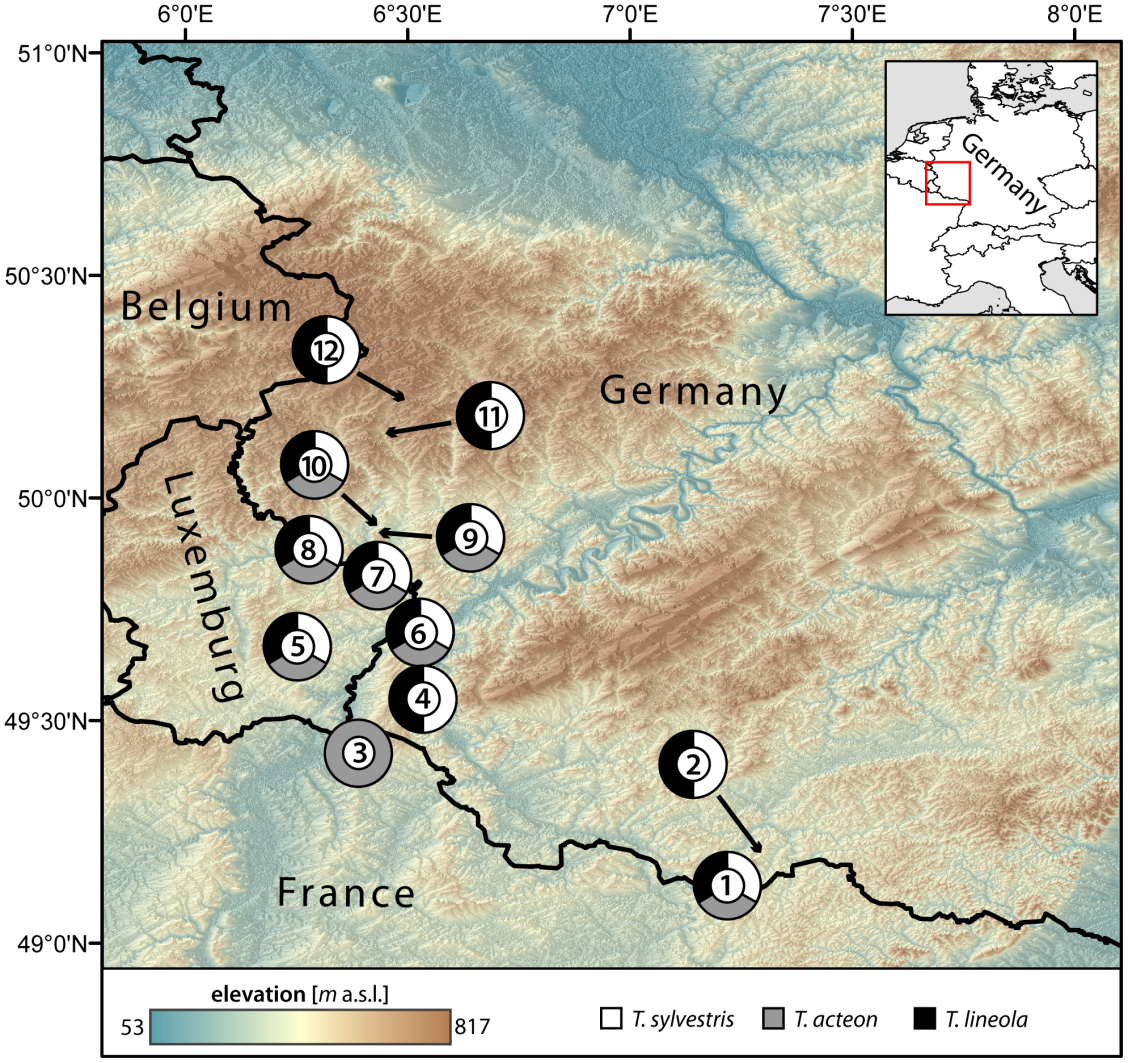
Locations of populations studied for all three *Thymelicus* species in southwestern Germany and adjoining areas in France and Luxemburg.

Our study area is located in the south-west of Germany and includes adjacent parts of France and Luxembourg (Fig. 1, Table S1). The sampling sites covered an area of approximately 120 km in north-south direction and 100 km in east-west direction. The landscape is characterised by a mosaic of residential areas, agricultural land, meadows, forests and semi-natural calcareous grasslands. Especially grasslands, but also some meadows and forest skirts provide suitable habitats for the three selected *Thymelicus* species, acting as valuable retreats and stepping stones [29].

The three selected model species *T. sylvestris*, *T. lineola* and *T. acteon* are closely related to each other with *T. lineola* and *T. acteon* being most distant related and where *T. sylvestris* clusters to a monophylum with *T. acteon* (Material S1). The three species show different habitat demands, even if they are co-occurring at suitable grassland patches: *T. lineola* occupies a broad ecological niche [30] and exhibits strong dispersal behaviour [31]. This combination of wide occurrence and strong dispersal behaviour results in a wide-spread, spatially continuous distribution. In contrast, *T. acteon* demands specific habitat characteristics like xerothermic climatic conditions and consequently occurs only in highly restricted, geographically disjunct calcareous grasslands. The third, intermediate species, *T. sylvestris* stands in-between both extremes, showing a broad ecological tolerance [30], similar to the generalist *T. lineola*, but shows a rather restricted dispersal behaviour [31].

### Molecular data and genetic cluster analysis

For our comparisons, we used a population genetic dataset based on 15 polymorphic allozymes published previously by [28] who did not account for landscape effects. Several studies have shown that the implications as drawn from allozymes and, where available, microsatellites loci were highly congruent in butterflies [32]–[34]. Here, the use of allozymes instead of other marker systems such as microsatellites has two advantages. 1) In Lepidopterans, locus-specific microsatellites are difficult to find and suitable polymorphic loci are consequently rare [35]–[38]. This is most likely due to almost identical flanking regions in the Lepidopteran microsatellite DNA [36], [39]. However, specificity of these regions is a crucial prerequisite for successful primer annealing [39]. 2) From a landscape genetic perspective, the use of potentially adaptive marker systems might be beneficial in the detection of spatial genetic differentiation in contrast to neutral marker systems, because spatial signals in markers under selection would appear more rapidly [40].

The data set comprised in total 1,063 individuals (417 *T. sylvestris*, 380 *T. lineola*, 160 *T. acteon*) sampled at 12 locations which were distributed across the same study area. Sample sizes ranged from 17 to 44 individuals per species and location. *Thymelicus sylvestris* and *T. lineola* were sampled at identical locations, while *T. acteon* was not found at four of the sampled locations and the data set was supplemented by one additional location (Fig. 1). The 15 enzyme systems provide the following 18 loci: MDH (2 loci), G6PDH, ACON, MPI, AAT (2 loci), FUM, PGI, ME, HBDH, APK, PGM, 6PGDH, IDH (2 loci), GPDH and PEP_Phe-Pro_. Most of these enzymes showed polymorphisms, except enzyme ME in *T. lineola* and GPDH in *T. sylvestris*. Details about the analytical procedure and the specific running conditions are given in [28]. We used the resulting dataset to estimate pairwise *F*_ST_ and *D*_est_ for each species in programmes Arlequin 3.1 [41] and smogd [42], respectively. The use of these two different measures of inter-population differentiation was recently recommended [43], because of the different underlying assumptions of either measure so that their combination might provide a more detailed impression into the underlying evolutionary processes of differentiation (see [43] and discussion in this study for further details). Tests for Hardy-Weinberg equilibrium and summary statistics for genetic diversity and differentiation were also calculated in Arlequin 3.1.

Prior to inferring landscape effects on genetic differentiation, the number of genetic groups (*K*) as well as their spatial delineation was evaluated for each species separately using the genetic clustering method implemented in the software Geneland [44]. This was necessary because (*i*) genetic differences can occur without any obvious landscape pattern (e.g. along secondary contact zones after postglacial expansion from distinct refugia or through anthropogenic introductions from another source population), which in turn would lead to (*ii*) erroneous conclusions on isolation-by-distance IBD/isolation-by-resistance IBR analyses on spatially independent structured data. Geneland assigns geo-referenced individuals to genetics clusters (*K*) that maximize Hardy-Weinberg-and Linkage-Equilibrium. *K* was treated as unknown to allow Geneland to vary *K* within a given range between 1 and the maximum number of populations depending on the species (i.e. 7 in *T. acteon* and 11 in both *T. sylvestris* and *T. lineola*). Markov Chains were run for 3,000,000 generations and sampled every 1000^th^ generation, after an initial burn-in of 300 samples after thinning (10%). Markov Chains with these settings were run 10 times independently and the iteration with the highest log posterior probability was chosen for inferring the most likely *K* and individual assignments.

### Modelling landscape effects on genetic differentiation

To test for landscape influences on genetic differentiation in each species, we modeled multiple species distribution models (SDM) incorporating topographic, bioclimatic and/or land use features. We then used resulting SDMs as resistance surfaces to derive inter-population connectivity estimates based on electrical circuit theory, and statistically compared these connectivity estimates to actual genetic differentiation. SDMs are increasingly applied for resistance surface parameterization in landscape genetic studies [23], [45] even under longer evolutionary time scales [46], [47], since they avoid the subjective parameterization of resistance surfaces which was criticized in the past [48].

### Species records

To model SDMs for the three *Thymelicus* species in the study area, presence data were taken from personal observations of JCH, D. Louy and T. Schmitt (Germany) covering the years 2003–2012. Further presence data were added from high resolution records downloaded from the GBIF database (www.gbif.org). The final datasets comprised 67 records for *T. sylvestris*, 62 for *T. lineola* and 28 records for *T. acteon*. Given their specific habitat demands and the sampling effort that was performed across the study area for either species (Fig. 1), we are confident to have compiled a representative sample that covered the realized distribution of the species in our study area.

### Environmental layers

For construction of the SDMs, we used freely available GIS based environmental layers. Bioclimatic data based on monthly averaged temperature and precipitation data with 30 arc seconds spatial resolution was obtained from the Worldclim Database (Vers. 1.4; www.worldclim.org; [49]). The comprehensive set of 19 bioclimatic variables are thought to be highly relevant for shaping species' Grinnellian (abiotic) niches [50]. In order to minimize the degree of inter-correlation among the variables (i.e. to keep pair-wise Pearson's R^2^<0.75), we selected a subset of variables (bio3, 7, 8, 9, 10, 11, 12, 15, 18, see Tab. 3/Appendix S3 for definitions) which were assumed to be most relevant for the study species. Topography-related data were derived from the SRTM Shuttle mission in 90 meters resolution (available through USGS seamless server; Table S2). Based on the altitude layers, we calculated slope and aspect using ArcGIS 9.3 (ESRI Redlands, California, USA). Finally, CORINE land use related data was obtained from the European Environmental Agency (www.eea.europa.eu). We either used CORINE 2006 data to assess current habitat availability as well as CORINE 1990 data for assessing recent land use changes. All environmental layers were re-sampled to uniform grid resolution of 90 m.

### Calculating the Potential Connectivity Model

We defined a set of hypotheses based on the available environmental data and generated five variable sets for comparing landscape effects on species-specific gene flow (therein called scenarios, Table S2). These scenarios represent various habitat characteristics (i.e. climate, topography and land use) that were found to be important for butterfly distributions at different spatial scales in previous studies [51]–[57]. Based on these variable sets and the respective species records, we computed species distribution models (SDMs) with the software Maxent 3.3.3e [58] to generate maps displaying habitat suitability for each species under a given scenario. As many other presence-pseudoabsence SDM algorithms, Maxent links environmental conditions at presence records of a given taxon to those environmental conditions available within a specific geographic area (background) to predict spatial patterns of environmental suitability. The SDM output represents the likelihood of species potential occurrence across a geographic area of interest (projection; for a detailed description see: [59]). We used Maxent instead of other available algorithms because it frequently outperforms other approaches [60]–[61], even if the number of presence locations is rather limited [62]–[63]. We ran Maxent with the default settings but used a bootstrap approach, which allows random selection of 70% of presence locations for model training and the remaining 30% for model testing. This procedure was repeated for 100 times and an averaged map of suitable habitats was generated across all repetitions. As output we selected the logistic format which ranges linearly from 0 (not suitable) to 1 (fully suitable). For model evaluation, the area under the receiver operating characteristic curve (AUC) was used [64]. In particular, the AUC as internally computed in Maxent is a measure for the ability of the model to distinguish the given presence records from the background data accounting for the proportion of the study area which is predicted to be suitable for the target species [58]. The AUC ranges between 0.5 (random prediction) to 1.0 (perfect discrimination between presence and pseudo-absence).

For the land use change scenario, we used land use data from CORINE 2006 as a categorical environmental layer - just as we had done for the land use scenario. However, we subsequently projected the model fit onto the CORINE 1990 layer to assess habitat change in terms of a stability surface. The stability surface is the average of both CORINE layers, with high values indicating suitable habitat patches that remain stable over the 16 years time span, whereas low values represent low habitat suitability, a strong habitat change in time, or both. This approach for calculating stability surfaces is commonly used to estimate land use change and habitat suitability across time (see [46]–[47] for examples).

The resulting SDMs were used as conductance surfaces (i.e. high values indicate good conductivity between two sites, whereas low values indicate poor conductivity [65]) in Circuitscape v.3.4.1 to calculate resistances to movement and gene flow among sampling locations [65]. Circuitscape is based on electrical circuit theory, which was recently adapted from electrical engineering for the assessment of landscape ecological questions [65]. In particular, Circuitscape defines nodes (grid cells) and associated unit resistors (the resistance value) that connecting two nodes and calculates resistance distances between focal locations based on a nodal analysis algorithm as described in [21]. As the habitat matrix had a very high extent (i.e. ∼7.6 Mio. cells), we chose a four-neighbor-connection scheme in order to meet the available computational capacities. It has been previously shown that four and eight-neighbor-connection scheme lead to highly similar outcomes [66].

### Comparing connectivity estimates with genetic data

Resulting resistance values among locations were statistically compared to estimates of genetic differentiation (i.e. *F*_ST_ and *D*_est_) using linear regression models as well as multiple regressions on distance matrices (MRDM) [67] in R v.2.14.1 [68]. For the linear regression models, the Akaike Information Criterion corrected for small sample sizes (AICc) was used for model comparisons within each species [69]. Despite their sensitivity for non-independence in pair-wise comparisons, multi-model inference based on information theory has been frequently applied in landscape genetic analyses [19]–[20] as the error entering the comparison was assumed to be equal for each model, which did not affects model ranking and thus still allows for assessing the relative model performance. To ascertain results obtained with the AIC model selection, we also estimated significance of MRDM models using 1,000 permutations. For MRDMs, the *ecodist* package for R was used [70].

## Results

### Genetic structures

No significant deviation from Hardy-Weinberg equilibrium was detected for any population in the respective species. Genetic diversity was comparatively low in *T. lineola* (mean ± SE; *AR* = 1.78±0.17 *H*_e_ = 9.6±2.1, *H*_o_ = 9.2±2.1), while *T. acteon* showed highest genetic diversities (*AR* = 1.88±0.18, *H*_e_ = 14.9±2.9, *H*_o_ = 12.5±2.6). *Thymelicus sylvestris* showed an intermediate level of genetic diversity, as compared to its congeners (*AR* = 1.80±0.10, *H*_e_ = 11.9±1.5, *H*_o_ = 11.0±1.4). The genetic differentiation was low in *T. lineola* (*F_st_* = 0.0081; *D_est_* = 0.0012; p = n.s.), while we detected highest genetic differentiation for *T. acteon* (*F_st_* = 0.0718; *D_est_* = 0.0143; p<0.0001). Again, *Thymelicus sylvestris* showed an intermediate level of genetic differentiation, with a rather low among-population variance (*F_st_* = 0.0179; *D_est_* = 0.0039; p<0.0001) (Table 1).

**Table 1:** Summary statistics for genetic diversity and differentiation for the three *Tymelicus* buttlerflies.

	*T. lineola*	*T. acteon*	*T. sylvestris*	*source*
AR	1.78±0.17	1.88±0.18	1.80±0.10	Louy *et al.* 2007
H_E_	9.6±2.1	14.9±2.9	11.9±1.5	Louy *et al.* 2007
H_O_	9.2±2.1	12.5±2.6	11.0±1.4	Louy *et al.* 2007
P_tot_	52.0±9.7	66.0±9.1	42.9±7.9	Louy *et al.* 2007
P_95_	36.4±9.4	49.3±13.4	32.3±4.2	Louy *et al.* 2007
F_st_	0.0081	0.0755	0.0179	Louy *et al.* 2007
D_est_	0.0012	0.0143	0.0039	Habel *et al.* 2013

### Genetic clustering results

The posterior density and log-likelihood levels of all Geneland runs stabilized long before the end of the Markov Chains, indicating that convergence was reached (Figure S1). For each of the species, all 10 replicate MCMC runs converged on K = 1 panmictic cluster (Appendix S4), indicating no absolute barriers affecting IBD or IBR assumptions.

### Species Distribution Models

**Figure 2:**
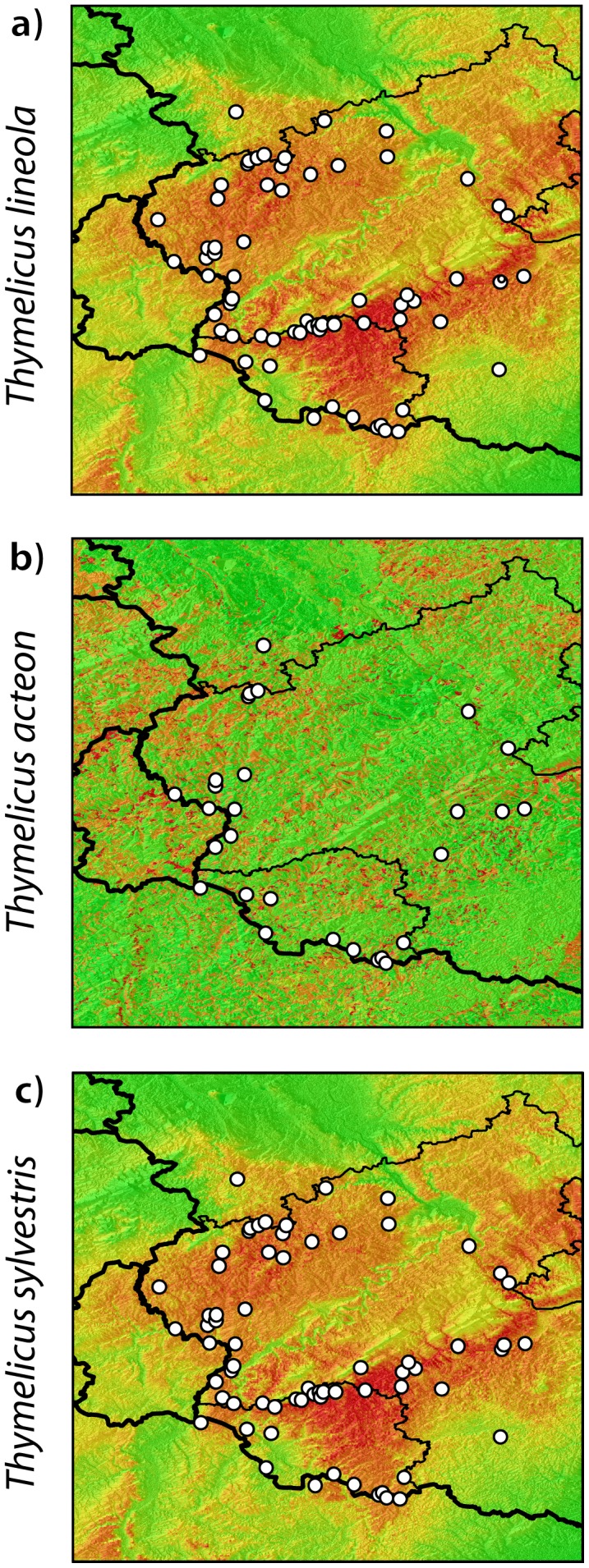
SDM output for *Thymelicus lineola* (A) *T. acteon* (B) and *T. sylvestris* (C) respectively. White circles on SDMs are presence locations used for modeling; Warmer colors (red) indicate higher suitability depending on the best model as presented in Table 2 (climate for *T. sylvestris*; land use change for *T. acteon*; note that *T. lineola* does not have a best model because of its panmictic state. Therefore, also climate is represented here).

AUC values derived from the SDMs ranged from ‘poor’ (AUC = 0.66, scenarios ‘land use’ and ‘land use change’ in *T. sylvestris*, Table 2) to ‘good’ (AUC = 0.86, scenario ‘all’ in *T. lineola*, Table 2) according to the classification scheme for model quality from [71] adapted from [64]. Variable contributions in multi-factorial SDMs (scenarios ‘climate’, ‘topography’ and ‘all’) differed between species (Table 3). For the topography scenario, *slope* contributed most to the SDM in all three species, followed by *aspect* and *altitude* (Table 3). In *T. acteon* a different set of variables had higher explanative power with respect to the climate scenario. Here, *precipitation of the warmest quarter* (bio18) was most important, followed by a set of temperature related variables (bio3, 7, 8, 9, 11; Table 3). In contrast, *Thymelicus lineola* and *T. sylvestris* had very similar variable contributions as a result of the highly similar distribution of occurrence records. In these species, the *mean temperature of the coldest quarter* followed by the *temperature annual range* contributed to more than half of the total model (Table 3). Finally, considering the entire predictor set, a combination of *slope* and *land use* contributed most in all species, but where *T. lineola* and *T. sylvestris* had again more similar variable contributions rather than *T. acteon* (Table 3). In accordance, *T. lineola* and *T. sylvestris* showed similar potential distributions containing large continuous areas of high suitability, whereas *T. acteon* shows a highly patchy distribution with large unsuitable areas surrounding potential habitat patches (Fig. 2).

**Table 2:** Comparison of the genetic structure in three *Thymelicus* butterflies with different landscape parameter sets.

		SDM	Linear regression model					MRDM	
Model F_st_		AUC	AICc	ΔAICc	ω	R^2^	p	R^2^	p
*T. lineola*									
	Fst∼distance	-	−321.65		0.21	−0.003	0.359	0.016	0.569
	Fst∼topography	0.76	−321.44	0.21	0.19	−0.007	0.424	0.012	0.603
	Fst∼climate	0.81	−321.41	0.23	0.19	−0.007	0.431	0.012	0.602
	Fst∼all	0.86	−320.87	0.77	0.14	−0.017	0.741	0.002	0.828
	Fst∼landusechange	0.68	−320.84	0.81	0.14	−0.018	0.795	0.001	0.854
	Fst∼landuse	0.67	−320.78	0.86	0.14	−0.019	0.893	0.000	0.926
*T. acteon*									
	**Fst∼landusechange**	**0.69**	**−94.70**		**0.56**	**0.202**	**0.009**	0.232	0.051
	**Fst∼landuse**	**0.71**	**−93.90**	**0.80**	**0.37**	**0.179**	**0.014**	0.209	0.069
	Fst∼distance	-	−88.12	6.58	0.02	−0.009	0.393	0.028	0.433
	Fst∼climate	0.79	−87.87	6.83	0.02	−0.018	0.476	0.020	0.748
	Fst∼topography	0.79	−87.44	7.26	0.01	−0.034	0.737	0.004	0.821
	Fst∼all	0.84	−87.41	7.29	0.01	−0.035	0.771	0.003	0.772
*T. sylvestris*									
	**Fst∼all**	**0.85**	**−273.53**		**0.67**	**0.252**	**<0.0001**	**0.266**	**0.002**
	**Fst∼climate**	**0.78**	−**271.89**	**1.65**	**0.29**	**0.229**	**<0.0001**	**0.244**	**0.010**
	Fst∼land use	0.66	−266.28	7.26	0.02	0.147	0.002	**0.162**	**0.024**
	Fst∼land use change	0.66	−265.52	8.02	0.01	0.135	0.003	**0.151**	**0.035**
	Fst∼distance	-	−263.78	9.75	0.01	0.107	0.009	0.123	0.068
	Fst∼topography	0.78	−262.73	10.81	0.00	0.09	0.015	0.106	0.102
Model D_est_									
*T. lineola*									
	**Dest∼distance**	**-**	**−806.68**		**0.48**	0.045	0.064	0.063	0.176
	Dest∼topography	0.76	−803.93	2.75	0.12	−0.004	0.373	0.015	0.559
	Dest∼climate	0.81	−804.10	2.58	0.13	−0.001	0.329	0.018	0.535
	Dest∼landusechange	0.68	−803.59	3.09	0.10	−0.010	0.493	0.009	0.647
	Dest∼all	0.86	−803.31	3.37	0.09	−0.015	0.652	0.004	0.780
	Dest∼landuse	0.67	−803.10	3.58	0.08	−0.019	0.951	0.000	0.968
									
*T. action*									
	**Dest∼landusechange**	**0.69**	**−274.21**		**0.45**	**0.159**	**0.021**	0.190	0.102
	**Dest∼landuse**	**0.71**	**−274.09**	**0.12**	**0.42**	**0.155**	**0.022**	0.186	0.090
	Dest∼climate	0.79	−269.81	4.40	0.05	0.015	0.244	0.052	0.608
	Dest∼distance	-	−268.92	5.29	0.03	−0.016	0.460	0.021	0.614
	Dest∼all	0.84	−268.41	5.79	0.02	−0.035	0.765	0.004	0.784
	Dest∼topography	0.79	−268.36	5.85	0.02	−0.037	0.845	0.001	0.893
									
*T. sylvestris*									
	**Dest∼climate**	**0.78**	**−723.08**		**0.45**	**0.099**	**0.011**	**0.115**	**0.049**
	**Dest∼land use**	**0.66**	**−721.12**	**1.96**	**0.17**	**0.066**	**0.033**	0.083	0.086
	**Dest∼land use change**	**0.66**	**−721.18**	**1.89**	**0.18**	**0.067**	**0.032**	0.084	0.085
	Dest∼distance	-	−720.50	2.57	0.12	0.055	0.046	0.073	0.118
	Dest∼all	0.85	−718.64	4.44	0.05	0.023	0.139	0.041	0.259
	Dest∼topography	0.78	−717.61	5.47	0.03	0.004	0.272	0.023	0.410

Genetic differentiation was inferred by *F*_ST_ (upper half) and *D*_est_ (lower half) respectively. SDM AUC values for each scenario (excepting classical IBD) showing the model quality are given as well as parameters for both, linear regression models and multiple regression based on distance matrices (MRDM). Bold values highlight models with highest support (ΔAICc<2 in combination with a significant R^2^ in linear regression models; significant R^2^ in MRDMs).

**Table 3:** Averaged variable contributions for the scenarios ‘topography’, ‘climate’ and ‘all’.

Scenario	Variable	*T. acteon*	*T. lineola*	*T. sylvestris*
Topography	alt	7.1	12.1	10.6
	aspect	21.1	29.7	32.3
	slope	71.8	58.2	57.1
Climate	bio3 (isothermality)	12.0	9.7	8.7
	bio7 (temperature annual range)	10.3	23.8	23.5
	bio8 (mean temperature of wettest quarter)	12.0	3.4	4.0
	bio9 (mean temperature of driest quarter)	11.3	10.1	10.0
	bio10 (mean temperature of warmest quarter)	3.6	4.6	5.7
	bio11 (mean temperature of coldest quarter)	16.1	32.0	31.4
	bio12 (annual precipitation)	5.2	10.0	11.3
	bio15 (precipitation seasonality)	5.4	3.3	3.0
	bio18 (precipitation of warmest quarter)	24.3	3.1	2.4
all	land use	37.7	24.9	23.2
	alt	1.3	8.1	7.7
	aspect	9.3	12.4	14.4
	slope	31.2	24.0	29.7
	bio3 (isothermality)	2.8	3.3	3.3
	bio7 (temperature annual range)	2.3	10.0	8.0
	bio8 (mean temperature of wettest quarter)	4.2	1.9	2.0
	bio9 (mean temperature of driest quarter)	1.0	0.1	0.3
	bio10 (mean temperature of warmest quarter)	0.1	0.7	0.4
	bio11 (mean temperature of coldest quarter)	2.1	6.5	4.7
	bio12 (annual precipitation)	2.1	5.8	4.6
	bio15 (precipitation seasonality)	1.5	1.1	0.8
	bio18 (precipitation of warmest quarter)	4.5	1.2	0.8

Note that land use dependent scenarios are not shown herein as they contain one single variable.

### Landscape effects of genetic differentiation

Results obtained with the various SDM-based connectivity estimates differed strongly among the three model species (Table 2). The generalist species *T. lineola* showed neither IBD nor any form of IBR using *F_st_* (max ΔAICc = 0.86). Using *D_est_*, the IBD scenario produced the best model (AICc = −806.68, ω = 0.48) however with a weak relationship (R^2^ = 0.045, p = 0.064). Furthermore, MRDM showed no landscape related signals for either estimate of genetic differentiation in *T. lineola*, suggesting that gene flow in this species is not affected by any spatial or landscape features at this scale. The most specialized species, *T. acteon* showed no significant IBD, but significant IBR for two scenarios (land use & land use change) with both *F_st_* and *D_est_* under multi-model inference. These signals become also prominent using MRDM for inference, even though models were slightly insignificant at p = 0.05 (land use change *F_st_*: R^2^ = 0.232, p = 0.051/*D_est_*: R^2^ = 0.190, p = 0.102). The combined results from AIC and MRDM suggest that land use and land use change both affect genetic differentiation among *T. acteon* populations. Genetic differentiation in *Thymelicus sylvestris* corresponded most strongly to the connectivity estimates derived from the SDM incorporating all variables (AICc = −271.89, ω = 0.67) using F_st_ and the information-theoretic approach. The climate related scenario was also within the most reliable models under AICc (ΔAICc = 1.65, ω = 0.29). However, MRDM suggested that land use and land use change were also important for explaining genetic differentiation in this species. The opposite becomes obvious using D_est_ as differentiation metric. Here, the information theoretic approach reveals climate, land use and land use change as highly informative, with climate being most important (AIC = −723.08, ω = 0.45). Surprisingly, the scenario covering the entire variable set contributed nearly no information (ΔAICc = 4.44, ω = 0.05). In addition, MRDM highlighted only climate as significantly related to genetic differentiation. In summary, the combined results of different differentiation metrics and inference methods suggest that the climatic conditions across the study site deliver the most important and stable relationship for adjusting gene flow in the intermediate species, with additional effects of land use. Classical IBD received less support against IBR models (Table 2, Figure S2) in all species. Interestingly, topography seems to play no role at all for any of the species.

## Discussion

Studying taxonomically related species inhabiting the same environment makes it possible to infer how species-specific ecological traits affect population genetic structuring without confounding effects of different landscapes or phylogenetic history [24]. By conducting a comparative landscape genetic study involving ecologically diverging Hesperid butterflies, we found different impacts of landscape parameters on the genetic structure of the three study species.

The obtained results show strong genetic differentiation and high genetic diversities in the specialist species *T. acteon*, and low genetic differentiation with accompanying low genetic diversities in the generalist species *T. lineola* with *T. sylvestris* standing in-between the two congeners. The amount of genetic diversity is typical for butterflies in this region (reviewed in [26]). Our analyses indicate that climate has a strong impact on the connectivity of *T. sylvestris* but that other variables (such as land use) might have become more influential in the most recent times. Land use as well as changes in land use patterns (i.e. assessed over a 16yr period) influences the connectivity of *T. acteon* populations. In contrast, *T. lineola* populations were panmictic, lacking any landscape related effects on genetic differentiation at this spatial scale.

### Diverging responses to identical landscape conditions

**Figure 3:**
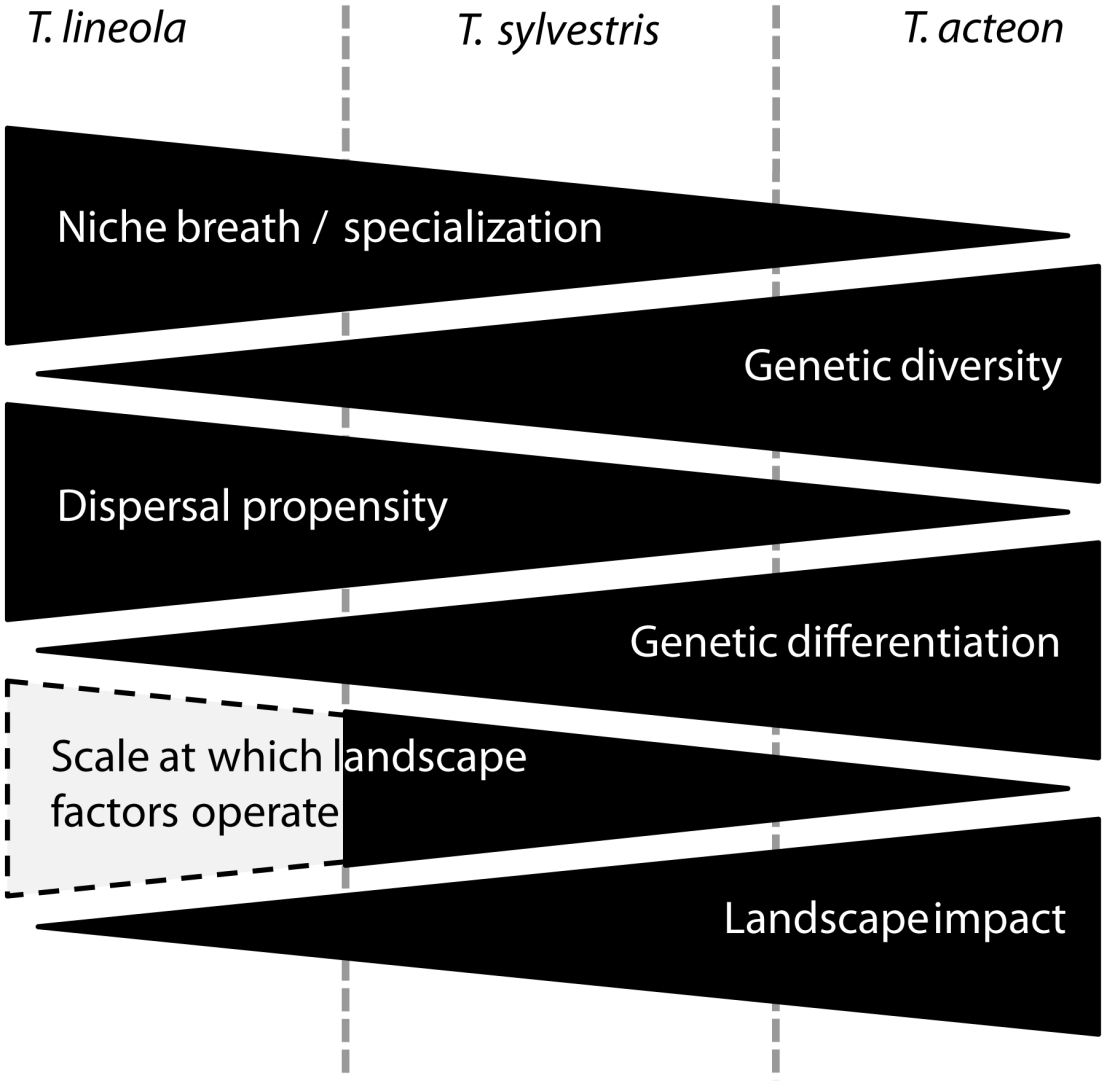
Schematic illustration about the gradual effects forcing on the three *Thymelicus* species. Hatched area highlights the hypothesized effect of landscape on gene flow in *T. lineola* on the macro-scale which was not testable in the study area.

Our data illustrate that closely related species representing a gradient of ecological traits (i.e. from generalist to specialist/from highly mobile to rather philopatric) also show a gradient of changing genetic structures and even more interesting of changing landscape genetic associations (Fig. 3). This highlights that ecological traits determine the species-specific resistance of the landscape matrix, so that its effect on population connectivity can differ strongly among closely-related species inhabiting the same landscape.

The strong genetic differentiation in *T. acteon* is concordant with its patchy occurrence predicted in our SDMs (Fig. 2b), which were best explained by the land-use parameters derived from the CORINE dataset. Furthermore, land-use related scenarios were the only ones that host an IBR-related signal among all competing scenarios in this species (Table 2). Here, the two scenarios ‘land-use’ and ‘land-use-change’ fit equally well, irrespective of the genetic differentiation metric or statistical inference method used. Thus, the landscape genetic signal in this specialist species is highly consistent among different analyses, leading to high certainty of inferences.

The slight differences between these two scenarios might be stochastic. However, since there is also consistence about the ranking across all approaches (i.e. land use change steadily explains slightly more variance under each situation than land use), land-use-change might be even more important, when addressing land-use-change over an even larger time period than the 16 years used here. Unfortunately, there is no information available to assess past land-use-changes covering this large geographical extent further into the past. Keeping time-lags between fragmentation and genetic responses accompanying these fragmentations in mind (e.g. as reviewed in [72]) there is some evidence that 16 years are not adequate to detect genetic impacts of altered habitats in this time period in a species with an annual generation time. Changes over this period result just in slightly different resistance surfaces between the scenarios ‘land-use’ and ‘land-use-change’. Nevertheless, *T. acteon* is becoming increasingly vulnerable in large parts of Europe [73] and has likely declined during the past 30 years within the study area due to habitat loss [29]. Thus, the slightly stronger signal of the land-use-change scenario in comparison to the land-use scenario might become even more prominent when extrapolating these changes further decades into the past, highlighting habitat loss as serve danger for this species.

The genetic diversities (such as heterozygosity or mean number of alleles) are highest in *T. acteon* compared to the other two species. This result is somewhat surprising, as the consequence of restricted gene flow and strong geographic restriction of local populations usually leads to rising genetic differentiation and declining genetic diversity, as frequently observed for species demanding specific habitat qualities and/or sedentary dispersal behaviour [10], [74]–[76]. However, there are also examples where genetic diversities in rare species exceed those of their common congeners [77]–[79]. This contrasting pattern to neutral genetic theory might be a result from hybridization ([80], but see [78]) or because of time-lags that display the past genetic diversity, when connectivity between populations was much higher than today [79], [81]. Indeed, genetic differentiation responds to habitat changes quicker than genetic diversity [82]–[83] so that the high genetic diversity observed for *T. aceton* may not yet reflect the negative consequences of on-going habitat alterations for this species.

In contrast to the specialist *T. acteon*, the generalist *T. lineola* represents opposing genetic features: the species shows a broad ecological amplitude and a much higher mobility [31]. This combination led to higher abundance pattern in combination with increased inter-population migration rates. These species traits lead to a rather panmictic genetic structure in our study area that appears to prevent landscape genetic relationships or IBD. This coherence between wide ecological amplitudes, high rates of individual exchanges (e.g. gene flow) and thus low genetic differentiation were frequently observed in other studies [84]–[85]. However, it needs to be considered that on a larger study extent, barriers such as oceans, large lakes, mountain ranges might become important for gene flow acting on a macro-scale [86]–[87]. The landscape matrix in our study area did not enable the assessment of such macro-scale effects, since the landscape matrix is rather continuous at this scale and large barriers are lacking, as indicated by the Geneland results.

Finally, the species standing in-between these two extremes, *T. sylvestris*, has an abundance like *T. lineola* but shows a sedentary dispersal behavior comparable to that of *T. acteon* [31]. The reduced dispersal propensity of this species coupled with its wide occurrence makes the colonization of a habitat nearby much more likely than of far distant habitats. Consequently, we obtain IBD and IBR signals for many sets of variables in this species (Table 2). However, when combining the information from the different assessment methods (*F_st_* vs. *D_est_*/multimodel inference vs. MRDM), landscape resistance based on the climate scenario was most important, delivering a consistent strong signal across the different inference methods used (Table 2, see also below). This contrasts to the IBR of *T. acteon*, where climate plays no role at all. In contrast to land-use, climate acts on a meso-scale at our study area (i.e. masking larger areas of the study extent rather than small habitat patches). In *T. sylvestris* the climate related SDM revealed high resistances along river valleys as well as on the higher elevations of the low mountain ranges (Fig 2c). These potential barriers act at a much larger scale and extent compared to the small and patchy habitat islands enclosed by more or less unfavourable habitats in *T. acteon.* Consequently, the different landscape features contributing to the IBR signals in these two species highlight the importance of scale and shape of the connective elements (or their respective barriers) in the landscape matrix where methodological shortcomings can be excluded (Engler, unpublished). However, the obtained IBR models explain only up to 24% of the variance in our dataset. That in turn indicates that the remaining variance of our data can only be explained by additional factors such as ecological traits and habitat requirements. These can be even more relevant for butterfly species than habitat size and habitat isolation, e.g. as shown for the Heath butterfly *Coenonympha tullia* [7]. Nevertheless, the extent of the relationships in our IBD/IBR comparisons are in concert with other studies [88] indicating that gene flow can be interpreted as an important component out of a variety of mechanisms influencing population genetic structure.

### Accounting for F_st_ and D_est_ in landscape genetic studies

Interestingly, in the case of *Thymelicus sylvestris*, the prominent signal under *F_st_* arising from the SDM using all landscape variables becomes completely eliminated when using *D_est_* as a differentiation metric. The fact that different metrics can lead to different conclusions is also evident in the ongoing debate about the utility of different genetic differentiation measures [89]–[94]. For example, traditional *F_st_* -like metrics are more sensitive to recent demographic changes (which depends e.g. on effective population size) than metrics which are independent of effective population size, such as *D_est_* [43], [90], [93]–[94]. This makes *F_st_* more sensible to effects of gene flow or drift in comparison to *D_est_*. Thus, from a landscape genetic perspective, using different types of differentiation metrics allows to test for the contribution of landscape effects in contemporary versus past times. If landscape composition change over time (and consequently the amount of gene flow mediated by the landscape), *F_st_* would respond much quicker to those changes while *D_est_* remains rather stable over time. In the case of *Thymelicus sylvestris,* this means that *D_est_* may highlight the landscape effect (here climate) of highest importance for gene flow in this species in former times, whereas *F_st_* highlights more recent landscape effects on genetic structure that involves also other landscape elements beside climate such as land use and topographical elements.

In contrast to the climate-only scenario, connectivity estimates involving all variables did not give highest importance to climatic factors. In particular, land use and slope contribute almost 54% of the total importance of this scenario, whereas the best performing variables from the climate scenario, bio11 (mean temp of coldest quarter) and bio7 (temp annual range) that contribute together 54.9%, contributing under the full model just 12.7% of the total importance. This might highlight the change of landscape factors important to gene flow in this species. As *T. sylvestris* is indeed common but not very mobile, anthropogenic land transformations of the past decades might now lead to a stronger fragmentation of populations which ultimately lead to changes in the contributions of landscape factors shaping gene flow as shown elsewhere [95]. Consequently, this might mean that this species is just at the tipping point of being of conservation concern (*sensu* [27]) where population trends swapping from stable to decreasing. Its congeners *T. acteon* and *T. lineola* showing both consistent results across the different metrics underpinning their stable state in terms of their abundance (insentinent and widespread vs. sensible and endangered) and specialization (generalist vs. specialist).

## Conclusions

Taxonomically close relatives serve as ideal model systems to study interspecific characteristics in ecological traits without confounding effects derived from different evolutionary histories. Yet, studies investigating the role of landscape on gene flow of closely related taxa inhabiting the same environment are still scarce. Our results reveal that even between sibling species, gene flow is affected by the landscape in very different ways. Thus, it is challenging to predict landscape genetic relationships in one species from a study involving another species, even if the two species are taxonomically closely related. Nevertheless, some generalizations are possible for specialist versus generalist species. In our study, the genetic structure of the generalist species with high dispersal propensities remained unaffected by the current landscape matrix, whereas specialist species were highly sensitive to fine scale habitat features. Changes of these features might therefore affect specialists more readily than generalist species with the negative consequences for their genetic setup. Species with an intermediate degree of specialization (here *T. sylvestris*) also interact with the landscape but at coarser scales in comparison to specialist species (here *T. acteon*). However, in light of global change such species might be on the highest risk due to negative genetic effects such as inbreeding depression, because changes in the habitat matrix can push former meta-population into isolated remnants [27]. This becomes also evident in *T. sylvestris* comparing the genetic structure under either *F_st_* or *D_est_*. Further studies focusing on the degree of habitat specialization in addition to dispersal capabilities are needed, ideally conducted with closely related taxa in other areas. Such comparative studies will greatly expand our current understanding of landscape genetic relationships and ultimately lead to more effective conservation and management of biodiversity.

## Supporting Information

Figure S1 Estimation of the number of panmictic clusters for each species. A) Convergence of the MCMC after thinning (see methods for details). Values prior to burn-in (indicated as red dashed line) were not considered as chain does not reached convergence. B) Frequency of the estimated number of populations along the chain after burn-in.
(DOC)

Figure S2 Scatterplots showing the differences of isolation by distance patterns with isolation by resistance patterns in the two species that show a spatial genetic structure (*Thymelicus sylvestris* is shown at the upper half, *T. acteon* at the lower half). Note that just the most prominent isolation by resistance pattern is shown (i.e. climate in *T. sylvestris* and land use change in *T. acteon*).
(DOC)

Table S1 Geographic coordinates of the sampling locations. ID numbers correspond to those stated in Fig. 1.
(DOC)

Table S2 Description of the landscape data used for resistance surface building depending on the scenario assumed. Note that the scenarios ‘land use’ and ‘land use change’ used the same data source. SDM refers to species distribution model.
(DOC)

Material S1 Evolutionary history of the three *Thymelicus* butterflies.
(DOC)
