# Supplementary material for: Comparative Landscape Genetics of Three Closely Related Sympatric Hesperid Butterflies with Diverging Ecological Traits
Source: PLoS One. 2014 Sep 3;9(9):e106526. doi: 10.1371/journal.pone.0106526 (PMC4153614; doi:10.1371/journal.pone.0106526)

**Figure S1.** Estimation of the number of panmictic clusters for each species. A) Convergence of the MCMC after thinning (see methods for details). Values prior to burn-in (indicated as red dashed line) were not considered as chain does not reached convergence. B) Frequency of the estimated number of populations along the chain after burn-in.


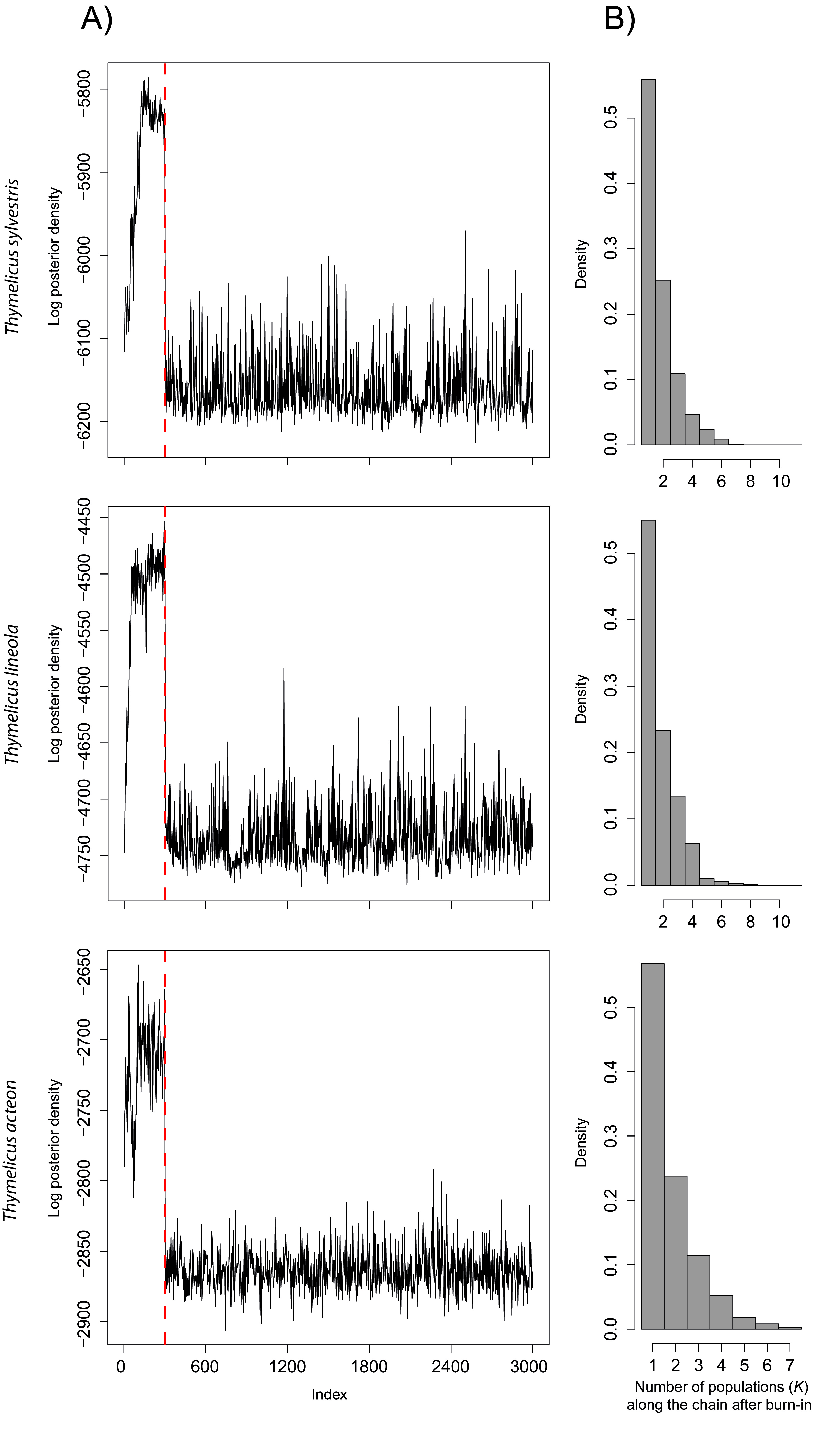

Supplement: Figure S1 — Estimation of the number of panmictic clusters for each species. A) Convergence of the MCMC after thinning (see methods for details). Values prior to burn-in (indicated as red dashed line) were not considered as chain does not reached convergence. B) Frequency of the estimated number of populations along the chain after burn-in. (DOC) [file pone.0106526.s001.doc]
