# Supplementary material for: Comparative Landscape Genetics of Three Closely Related Sympatric Hesperid Butterflies with Diverging Ecological Traits
Source: PLoS One. 2014 Sep 3;9(9):e106526. doi: 10.1371/journal.pone.0106526 (PMC4153614; doi:10.1371/journal.pone.0106526)

**Figure S2.** Scatterplots showing the differences of isolation by distance patterns with isolation by resistance patterns in the two species that show a spatial genetic structure (*Thymelicus sylvestris* is shown at the upper half, *T. acteon* at the lower half). Note that just the most prominent isolation by resistance pattern is shown (i.e. climate in *T. sylvestris* and land use change in *T. acteon*).


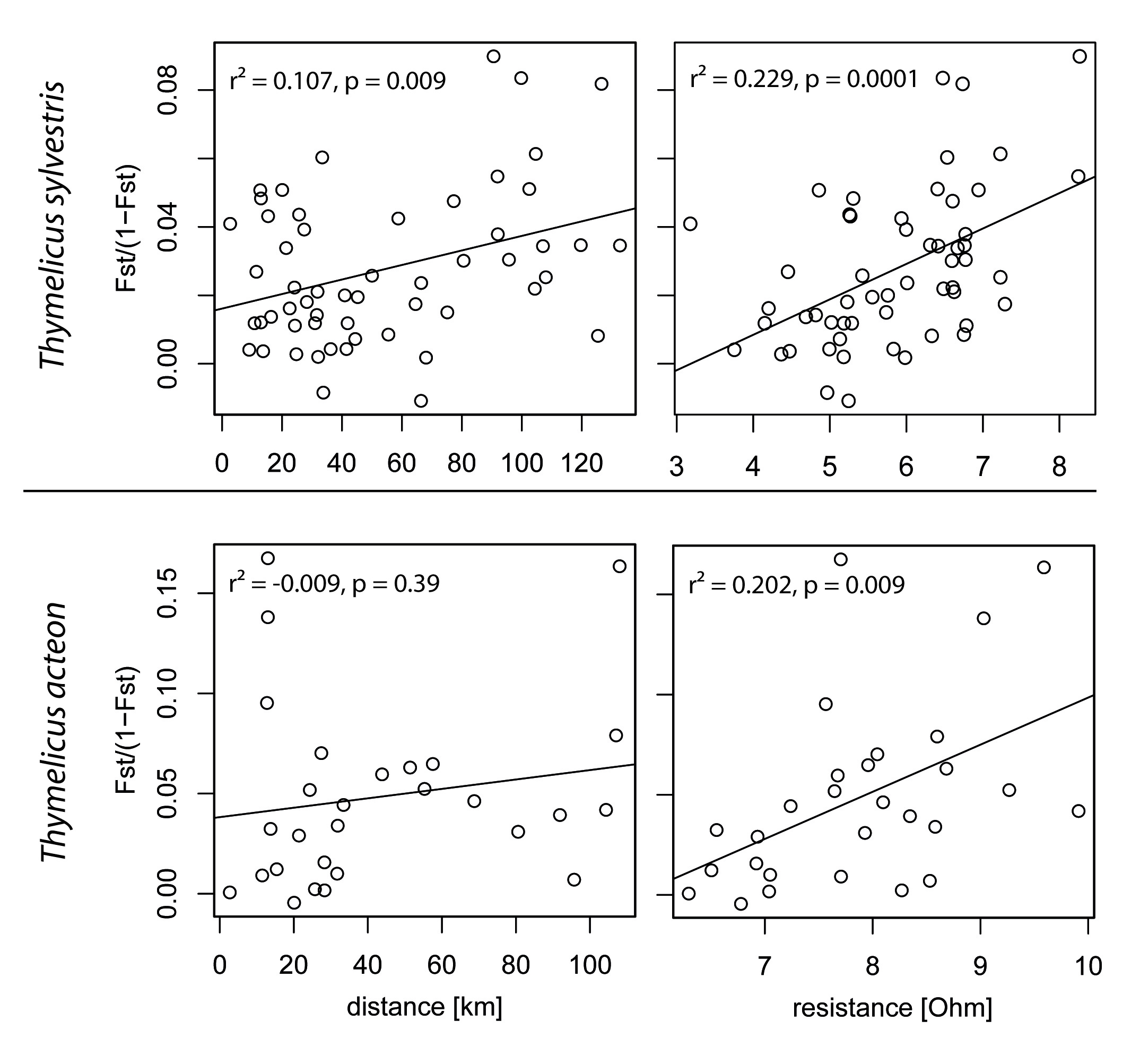

Supplement: Figure S2 — Scatterplots showing the differences of isolation by distance patterns with isolation by resistance patterns in the two species that show a spatial genetic structure (Thymelicus sylvestris is shown at the upper half, T. acteon at the lower half). Note that just the most prominent isolation by resistance pattern is shown (i.e. climate in T. sylvestris and land use change in T. acteon). (DOC) [file pone.0106526.s002.doc]
