# Supplementary material for: Comparative Landscape Genetics of Three Closely Related Sympatric Hesperid Butterflies with Diverging Ecological Traits
Source: PLoS One. 2014 Sep 3;9(9):e106526. doi: 10.1371/journal.pone.0106526 (PMC4153614; doi:10.1371/journal.pone.0106526)
Supplement: Table S1 — Geographic coordinates of the sampling locations. ID numbers correspond to those stated in Fig. 1. (DOC) [file pone.0106526.s003.doc]

**Table S1.** Geographic coordinates of the sampling locations. ID numbers correspond to those stated in Fig. 1.

| **Location – ID** | **lat** | **lon** | ***T. sylvestris*** | ***T. lineola*** | ***T. acteon*** |
| --- | --- | --- | --- | --- | --- |
| Niedergailbach – 1 | 49.128 | 7.220 | x | x | x |
| Mimbach/Badstube - 2 | 49.212 | 7.296 | x | x |  |
| Montenach - 3 | 49.425 | 6.388 |  |  | x |
| Eiderberg/Freudenburg - 4 | 49.549 | 6.537 | x | x |  |
| Niederanven - 5 | 49.664 | 6.252 | x | x | x |
| Wasserliesch - 6 | 49.696 | 6.527 | x | x | x |
| Echternacherbrück - 7 | 49.819 | 6.428 | x | x | x |
| Ourtal - 8 | 49.882 | 6.275 | x | x | x |
| Römersköpfchen - 9 | 49.921 | 6.446 | x | x | x |
| Bettingen - 10 | 49.942 | 6.427 | x | x | x |
| Schönecken - 11 | 50.144 | 6.462 | x | x |  |
| Weinsheim/Prüm - 12 | 50.224 | 6.488 | x | x |  |
