# Supplementary material for: Comparative Landscape Genetics of Three Closely Related Sympatric Hesperid Butterflies with Diverging Ecological Traits
Source: PLoS One. 2014 Sep 3;9(9):e106526. doi: 10.1371/journal.pone.0106526 (PMC4153614; doi:10.1371/journal.pone.0106526)
Supplement: Table S2 — Description of the landscape data used for resistance surface building depending on the scenario assumed. Note that the scenarios ‘land use’ and ‘land use change’ used the same data source. SDM refers to species distribution model. (DOC) [file pone.0106526.s004.doc]

**Table S2.** Description of the landscape data used for resistance surface building depending on the scenario assumed. Note that the scenarios ‘land use’ and ‘land use change’ used the same data source. SDM refers to species distribution model.

| **Scenario** | **Description** | **Data source** |
| --- | --- | --- |
| **Distance** | Classical isolation-by-distance measured as straight distance between coordinate pairs. | - |
| **Topography** | SDM was computed on three topographical variables: altitude, aspect & slope. | SRTM Shuttle mission available through: http://earthexplorer.usgs.gov |
| **Climate** | This SDM incorporates 9 out of 19 bioclimatic variables describing the climatic conditions at each location with respect to species' demands. These are: bio3 (Isothermality), bio7 (Temperature annual range), bio8 (mean temperature of wettest quarter), bio9 (mean temperature of driest quarter), bio10 (mean temperature of warmest quarter), bio11 (mean temperature of coldest quarter), bio12 (annual precipitation), bio15 (precipitation seasonality) and bio18 (precipitation of warmest quarter). | Available through: http://worldclim.org |
| **Land use /  Land use change** | Data for these two SDMs derived from the CORINE Landcover data based on 2006 (land use) and 1990 (projected onto for land use change) landcover. | Available through: http://www.eea.europa.eu |
| **all** | Combining topography, climate and land use datasets to assess the impact of all landscape factors in concert. |  |
