# Supplementary material for: Comparative Landscape Genetics of Three Closely Related Sympatric Hesperid Butterflies with Diverging Ecological Traits
Source: PLoS One. 2014 Sep 3;9(9):e106526. doi: 10.1371/journal.pone.0106526 (PMC4153614; doi:10.1371/journal.pone.0106526)
Supplement: Material S1 — Evolutionary history of the three Thymelicus butterflies. (DOC) [file pone.0106526.s005.doc]

**Material S1.** Evolutionary history of the three *Thymelicus* butterflies.

To assess the phylogenetic relationships between the three *Thymelicus* species, we compiled a phylogenetic analysis based on a 658 bp fragment of the mitochondrial COI gene. We conducted a GenBank query under http://www.ncbi.nlm.nih.gov/genbank and selected nucleotide sequences of two specimens for each species as well as choosing one specimen (*Hesperia comma*) as outgroup (Appendix S2 Table 1; Hausmann *et al*. 2011). Sequences were aligned and uncorrected genetic distances (p-distance) between and among species were calculated using the programme MEGA 5.05 (Appendix S2 Table 2; Kumar *et al.* 2008; Tamura *et al.* 2011).

For reconstructing the evolutionary history in *Thymelicus*, a consensus tree based on the Neighbor-Joining method (Saitou & Nei 1987) was computed in MEGA and inferred using 1000 bootstrap replicates (Felsenstein 1985). The evolutionary distances underlying this consensus tree were computed using the Maximum Composite Likelihood method (Tamura *et al.* 2004) with transitions, transversions and all three codon-positions included in the analysis (Appendix S2 Fig. 1).


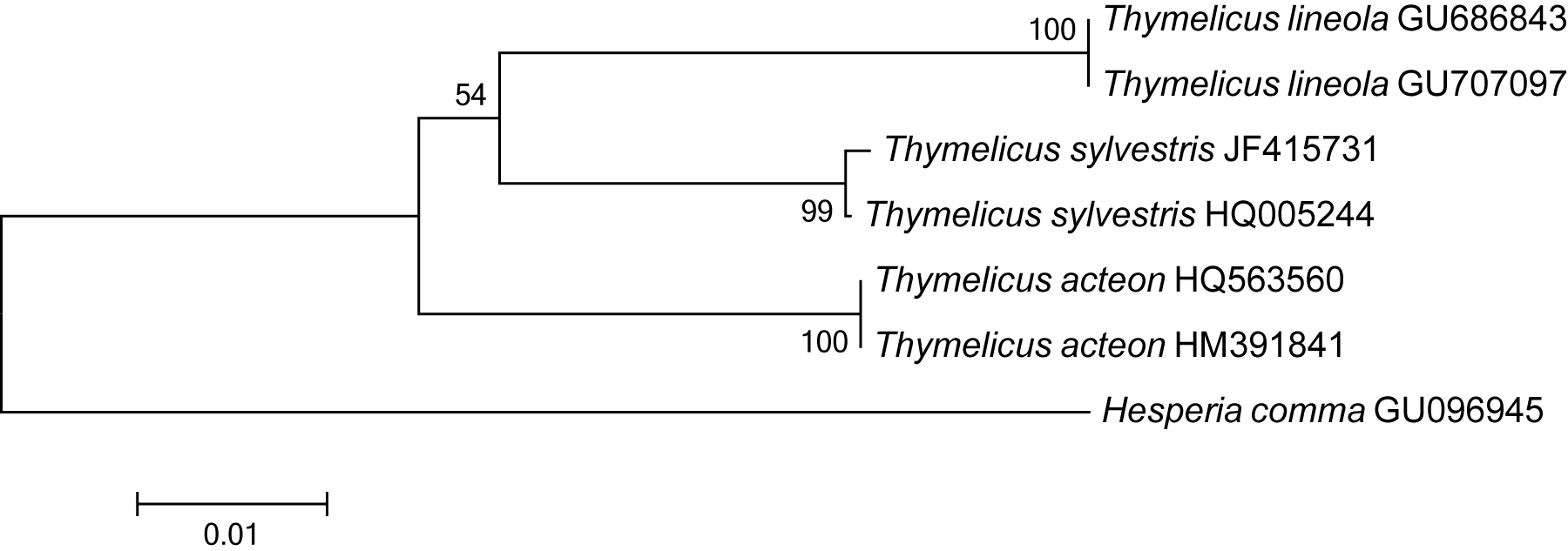


**Material S1 Fig. 1.** Consensus tree inferred by using the Neighbor-Joining method accounting for the fifty percent majority-rule. Numbers next to the branches correspond to the bootstrap support (1000 replicates). *Hesperia comma* was used as outgroup taxon.

The generalist *Thymelicus lineola* is most distant related to the specialist species *T. acteon* (5.02 % of substitutions). *Thymelicus* *sylvestris*, the ecologically intermediary species clusters together with *T. acteon* to a monophylum with a p-distance of 4.3% of substitutions. *Thymelicus lineola* thus represents the paraphylum to the other two species because of a slightly higher genetic distance to *T. sylvestris* (4.5%).

**Material S1 Table 1. Species and GenBank accession numbers of the individuals used for estimating genetic distance between species.**

| **Species** | **GenBank accession no.** |
| --- | --- |
| *Thymelicus lineola* | GU686843 |
| *Thymelicus lineola* | GU707097 |
| *Thymelicus sylvestris* | JF415731 |
| *Thymelicus sylvestris* | HQ005244 |
| *Thymelicus acteon* | HQ563560 |
| *Thymelicus acteon* | HM391841 |
| *Hesperia comma* | GU096945 |

**Material S1 Table 2.** Uncorrected pairwise genetic distance (average %) of the COI sequences within (diagonal) and between (below diagonal) species of the genus *Thymelicus*.

|  | *T. acteon* | *T. lineola* | *T. sylvestris* |
| --- | --- | --- | --- |
| *Thymelicus acteon* (n = 2) | 0.00 |  |  |
| *Thymelicus lineola* (n = 2) | 5.02 | 0.00 |  |
| *Thymelicus sylvestris* (n = 2) | 4.30 | 4.50 | 0.15 |

References Material S1

Hausmann, A., Haszprunar, G., Gegerer, A.H., Speidel, W., Behounek, G. & Herbert, P.D.N. (2011) Now DNA-barcoded: the butterflies and larger moths of Germany. *Spinxia*, **34**, 47-58.

Kumar, S., Nei, M., Dudley, J., & Tamura, K. (2008) MEGA: a biologist-centric software for evolutionary analysis of DNA and protein sequences. *Briefings in bioinformatics*, **9**, 299-306.

Saitou, N. & Nei, M. (1987) The neighbor-joining method: A new method for reconstructing phylogenetic trees. *Molecular Biology and Evolution*, **4**, 406-425.

Felsenstein, J. (1985). Confidence limits on phylogenies: An approach using the bootstrap. *Evolution*, **39**, 783-791.

Tamura, K., Nei, M. & Kumar, S. (2004) Prospects for inferring very large phylogenies by using the neighbor-joining method. *Proceedings of the National Academy of Sciences USA*, **101**, 11030-11035.

Tamura, K., Peterson, D., Peterson, N., Stecher, G., Nei, M. & Kumar S. (2011) MEGA5: Molecular Evolutionary Genetics Analysis using Maximum Likelihood, Evolutionary Distance, and Maximum Parsimony Methods. *Molecular Biology and Evolution*, **28**, 2731-2739.
